# Osteoporosis treatment gap prior to femoral fracture and prevalence of pharmacological risk factors: a prospective observational study

**DOI:** 10.1007/s00402-026-06401-5

**Published:** 2026-07-03

**Authors:** Lukas Böck, Katharina Kerschan-Schindl, Martin Frossard, Stefan Hajdu, Richard Crevenna, Martina Anditsch, Gunar Stemer, Anna Antoni

**Affiliations:** 1 Department of Hospital Pharmacy, University Hospital Vienna, Vienna, Austria; 2 Department of Physical Medicine, Rehabilitation and Occupational Medicine, Medical University of Vienna, Vienna, Austria; 3 Department of Orthopedics and Trauma Surgery, Medical University of Vienna, Vienna, Austria; 4 Department of Hospital Pharmacy, Hanusch Hospital, Vienna, Austria

**Keywords:** Osteoporosis, Fragility fracture, Guideline adherence, Fall-risk-increasing drugs, Femoral fracture

## Abstract

**Introduction:**

Hip fractures are a major complication of osteoporosis and are associated with high morbidity, mortality, and costs. Despite clear guideline recommendations, pharmacological osteoporosis treatment remains underutilized. In addition, medications that increase fall and fracture risk further contribute to fracture occurrence. The aim of this study was to assess guideline concordance of osteoporosis therapy and to quantify the prevalence of medications associated with increased fall and fracture risk prior to the fracture in patients admitted with femoral fractures.

**Materials and methods:**

In this prospective observational study, 145 patients aged ≥ 55 years admitted with femoral neck, per- and subtrochanteric, femoral shaft and distal femur fractures to a tertiary academic trauma center between April and June 2025 were included. Pre-admission medication was assessed using structured medication reconciliation. Guideline concordance of pre-fracture osteoporosis therapy was evaluated according to the current Austrian osteoporosis guideline (Dimai et al., 2024). Fall-risk-increasing drugs (FRIDs) were identified using the STOPPFall criteria, and fracture-associated medications were recorded based on Austrian osteoporosis guideline-defined drug classes.

**Results:**

Among 145 included patients (mean age 80.4 years; 71% female), 117 (81%) met criteria for pharmacological osteoporosis treatment prior to the fracture, defined by a preexisting osteoporosis diagnosis or a 10-year fracture risk above the treatment intervention threshold using the FRAX^®^ calculator. Only 9 (7.7%) of these patients were treated in accordance with guideline recommendations. A treatment gap was observed in 78.6% of therapy-eligible patients. At least one FRID was prescribed in 70% of patients, and 57% received at least one medication associated with increased fracture risk.

**Conclusions:**

Patients presenting with femoral fractures demonstrate a substantial gap in guideline-concordant osteoporosis therapy and a high prevalence of medications associated with increased fall and fracture risk. These findings highlight important opportunities for systematic primary and secondary fracture prevention and structured medication review.

## Introduction

Hip fractures represent one of the most severe consequences of osteoporosis and are associated with substantial morbidity, loss of independence, and increased mortality in older adults. One-year mortality after hip fracture approaches 20%, and survivors remain at increased risk for subsequent fragility fractures [1]. As a major osteoporotic fracture, a hip fracture reflects underlying osteoporosis and therefore constitutes a critical, time-sensitive window for intervention, requiring prompt initiation of evidence-based secondary fracture prevention irrespective of bone mineral density [2].

The epidemiological impact of osteoporosis extends beyond individual fracture events. Across Europe, approximately 32 million people are affected, resulting in considerable healthcare costs and fracture-related mortality. Although hip fractures represent a minority of osteoporotic fractures, they account for a disproportionate share of fracture-related costs and mortality [3]. Austria ranks among the European countries with the highest age-standardized hip fracture incidence and lifetime fracture probability. Despite this substantial disease burden and the availability of effective pharmacological therapies, significant care gaps persist. Previous Austrian data have demonstrated low rates of osteoporosis treatment even after fragility fractures, including reports that most patients remain untreated following hip fracture [4, 5]. These findings indicate that the treatment gap remains considerable both across Europe and in Austria.

The recently updated Austrian osteoporosis guideline (Dimai et al., 2024) emphasizes a risk-based approach to treatment initiation, incorporating clinical risk assessment tools such as FRAX^®^, which was updated and validated for Austria based on national fracture data in 2022. The guideline further allows treatment decisions independent of bone mineral density in high-risk individuals, particularly after hip or vertebral fragility fractures, and highlights the importance of timely secondary fracture prevention following major osteoporotic fractures [2].

Nevertheless, a persistent and well-documented gap exists between guideline recommendations and real-world clinical practice. Numerous studies across Europe and North America have demonstrated that a large proportion of patients with osteoporotic fractures do not receive appropriate pharmacological osteoporosis treatment, even when clear indications are present. This treatment gap remains pronounced after hip fractures, which represent both a sentinel event and a critical opportunity for secondary fracture prevention [6, 7].

In addition to the underuse of osteoporosis therapy, medication-related risk factors - particularly fall-risk-increasing drugs (FRIDs) - represent an important and potentially modifiable contributor to fracture risk in older adults. Several drug classes are associated with sedation, orthostatic hypotension, impaired balance or cognitive dysfunction and thereby increase the likelihood of falls. Furthermore, certain medications negatively affect bone metabolism or are independently associated with an increased fracture risk [8].

Current international guidelines therefore emphasize structured medication reviews as an essential component of fracture prevention strategies in older patients. Tools such as the STOPPFall criteria provide evidence-based frameworks for identifying potentially inappropriate medications associated with increased fall risk [9]. However, data on the prevalence of FRIDs and fracture-associated medications in patients presenting with hip fractures remain limited, particularly when assessed in conjunction with the adequacy of osteoporosis treatment.

Importantly, many existing studies focus on post-fracture treatment initiation or rely on administrative prescription data, while detailed analyses of pre-admission medication regimens at the time of hospital admission are less frequently reported [10]. This aspect is clinically relevant, as the pre-fracture medication profile reflects long-standing prescribing patterns and represents a critical target for intervention during acute hospital care. In addition, only limited data are available that combine a guideline-based assessment of osteoporosis therapy with an evaluation of medications associated with increased fall and fracture risk in the same patient population.

Therefore, the aim of the present prospective observational study was to evaluate the guideline concordance of osteoporosis therapy in patients admitted with hip fractures to a tertiary trauma center. In addition, we sought to quantify the prevalence of medications associated with increased fall risk and increased fracture risk at the time of hospital admission.

## Methods

### Study design and setting

This prospective observational study was conducted at a tertiary academic trauma center. Patients were enrolled over a three-month period between April and June 2025. As the study aimed to evaluate pre-fracture osteoporosis management, treatment indication and guideline concordance were assessed based on the clinical status prior to the index fracture event, independent of the fracture itself.

The study was approved by the local ethics committee (EK 1138/2025). The study was performed in accordance with the ethical standards as laid down in the 1964 Declaration of Helsinki and its later amendments.

### Study population

Patients aged 55 years or older admitted with femoral fractures were eligible for inclusion. Femur fractures were defined according to ICD-10 codes S72.0–S72.4. While most cases represented proximal femur fractures (femoral neck, per- and subtrochanteric femur fracture), a small proportion of femoral shaft and distal femur fractures was also included. Only low-energy trauma (fragility) fractures were included. The patients were treated according to the standard protocol of the trauma department. Patients were excluded if complete pre-admission medication data were unavailable.

### Data collection

Patients were identified through the hospital information system. Pre-admission medication was assessed using structured medication reconciliation, including review of medical records, electronic health records, and patient interviews. Demographic data, comorbidities, fracture type, and documented osteoporosis diagnoses were recorded. Data collection and patient interviews were performed by a senior clinical pharmacist.

### Assessment of osteoporosis treatment and guideline concordance

**Fig. 1:**
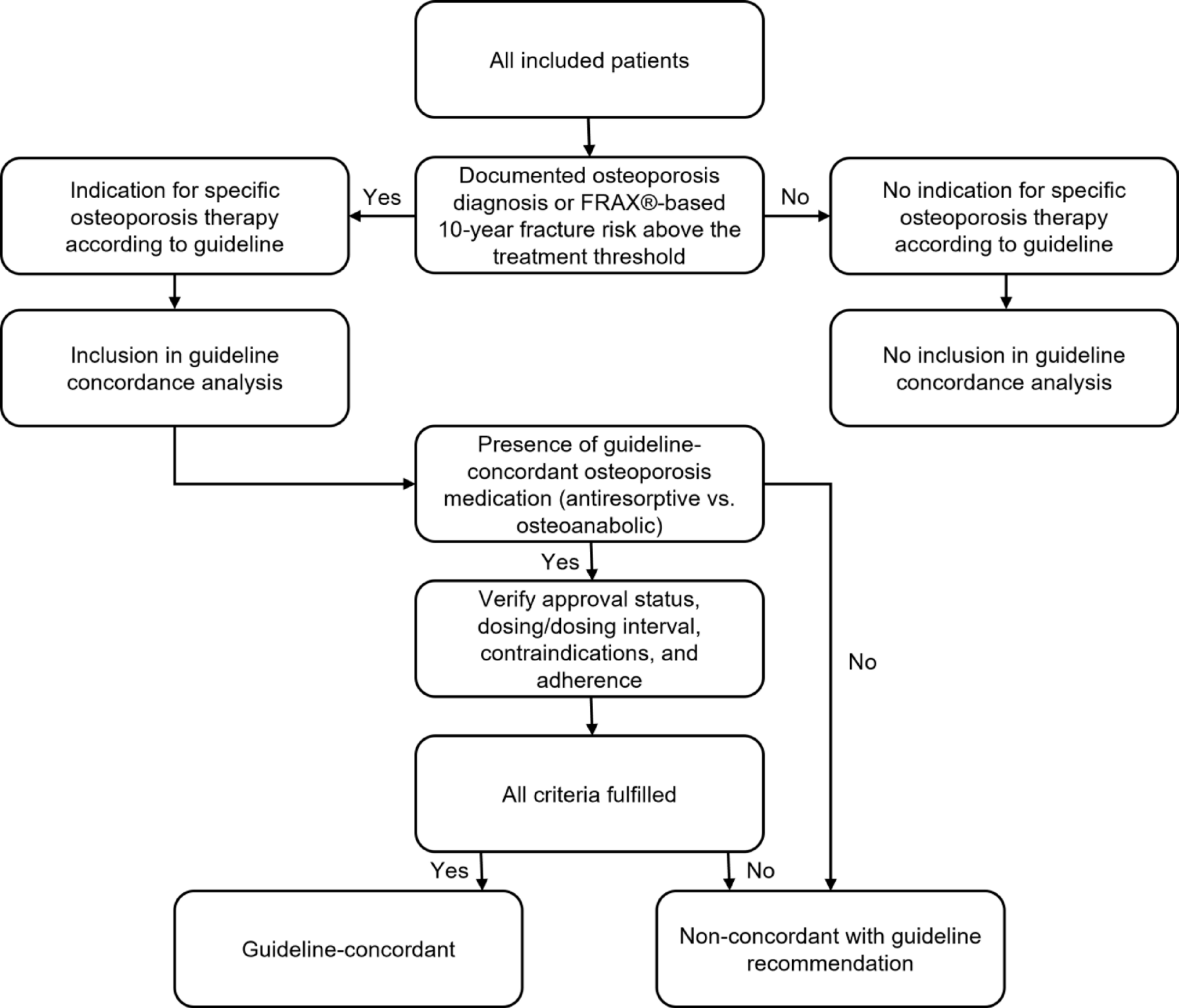
Flowchart of the guideline concordance assessment of pre-admission osteoporosis therapy

Guideline concordance of osteoporosis therapy was evaluated according to the current guidelines of the Austrian Society for Bone and Mineral Metabolism (Dimai et al., 2024) [2]. Patients were considered eligible for pre-fracture pharmacological osteoporosis treatment if they had a documented osteoporosis diagnosis or a high or very high 10-year fracture risk as assessed by the FRAX^®^ tool. Cases without an existing indication according to the guideline were excluded from the concordance analysis, as drug therapy was not required in these situations. In addition, it was examined whether the prescribed drug therapy was consistent with the guideline’s recommendations regarding the following aspects: (1) appropriate choice of pharmacological agent according to individual fracture risk (osteoanabolic vs. antiresorptive therapy), (2) correct dosing and administration in accordance with the respective prescribing information, (3) absence of relevant contraindications as defined in the prescribing information, and (4) adequate adherence based on patient interviews. The assessment of guideline concordance is illustrated in Fig. 1.

Contraindications included, among others, severe renal impairment for bisphosphonates, hypocalcemia, or other drug-specific safety considerations as specified in the respective product information.

### Assessment of fall- and fracture-associated medications

FRIDs were identified using the STOPPFall criteria. The STOPPFall criteria were developed by the European Geriatric Medicine Society (EuGMS) and consist of 14 evidence-based medication classes associated with increased fall risk in older adults, intended to support structured medication review and deprescribing strategies. Medications associated with increased fracture risk were recorded based on guideline-defined drug classes known to affect bone metabolism or fracture risk.

### Statistical analysis

Data were analyzed descriptively. Categorical variables are presented as frequencies and percentages, and continuous variables as means with standard deviations.

## Results

### Study population

A total of 145 patients were included. The mean age was 80.4 years, and 71% were female (Table 1). Among the included patients, 117 (81%) met pre-fracture criteria for pharmacological osteoporosis treatment based on guideline recommendations. The remaining 28 patients (19%) did not meet treatment criteria, as they had neither a documented osteoporosis diagnosis nor a high or very high fracture risk according to FRAX^®^ assessment prior to the fracture.

**Table 1:** Baseline characteristics of the study population

Variable	Total (*n* = 145)	Subgroup with guideline assessment (*n* = 117)
Age, years	80.4 ± 9.2	82.3 ± 7.9
Minimum	55	59
Maximum	97	97
Sex, n (%)		
Female	103 (71.0)	92 (78.6)
Male	42 (29.0)	25 (21.4)
Comorbidities, n (%)		
Hypertension	95 (65.5)	82 (70.1)
Visual impairment	4 (2.8)	4 (3.4)
Cardiovascular disease	65 (44.8)	55 (47.0)
Parkinson’s disease	7 (4.8)	4 (3.4)
Dementia	28 (19.3)	27 (23.1)
Diabetes mellitus	30 (20.7)	27 (23.1)
Hyperlipidemia	60 (41.4)	51 (43.6)
Chronic obstructive pulmonary disease	22 (15.2)	19 (16.2)
Malignancy	21 (14.5)	17 (14.5)
Prior osteoporosis diagnosis	41 (28.3)	41 (35.0)
FRAX® risk category, n (%)		
Low	17 (11.7)	1 (0.9)
Moderate	16 (11.0)	4 (3.4)
High	28 (19.3)	28 (23.9)
Very high	84 (57.9)	84 (71.8)
Vitamin D and/or calcium supplementation, n (%)	73 (50.3)	69 (59.0)
Fracture type (ICD-10), n (%)		
Femoral neck fracture (S72.0)	74 (51.0)	61 (52.1)
Pertrochanteric fracture (S72.1)	51 (35.2)	40 (34.2)
Subtrochanteric fracture (S72.2)	1 (0.7)	1 (0.9)
Femoral shaft fracture (S72.3)	15 (10.3)	13 (11.1)
Distal femur fracture (S72.4)	4 (2.8)	2 (1.7)

Values are presented as mean ± standard deviation or number (percentage)

### Guideline concordance of osteoporosis therapy

Among the 117 therapy-eligible patients, only 9 (7.7%) received osteoporosis treatment fully concordant with guideline recommendations, while 108 (92.3%) showed deviations from the guideline.

A specific osteoporosis medication was prescribed in 25 patients (21.4%). Of these, 16 (64.0%) were classified as non-concordant, and 9 (36.0%) as fully guideline-concordant. Denosumab was the most frequently prescribed agent (*n* = 14), followed by ibandronate (*n* = 4), teriparatide (*n* = 3), and alendronate (*n* = 2). Other agents, including risedronate and zoledronate, were used only rarely (*n* = 1 each), while no prescriptions of abaloparatide, romosozumab, hormone replacement therapy, or raloxifene were observed.

Overall, 92 of 117 patients did not receive any specific osteoporosis medication despite a pre-fracture treatment indication, corresponding to a treatment gap of 78.6%.

The main reasons for non-concordance were lack of treatment despite an indication (*n* = 90, 83.3%), inappropriate choice of medication (*n* = 14, 13.0%), and insufficient treatment adherence (*n* = 5, 4.6%).

In a subgroup analysis of 41 patients with a documented diagnosis of osteoporosis prior to the fracture, 23 (56.1%) received specific pharmacological therapy, while 18 (43.9%) did not. Calcium and/or vitamin D supplementation was reported in 32 patients (78.0%). Among the 18 untreated patients, 16 cases (88.9%) were due to lack of treatment despite indication, and 2 cases (11.1%) were attributed to poor adherence.

### Prevalence of fall-risk-increasing drugs

**Fig. 2:**
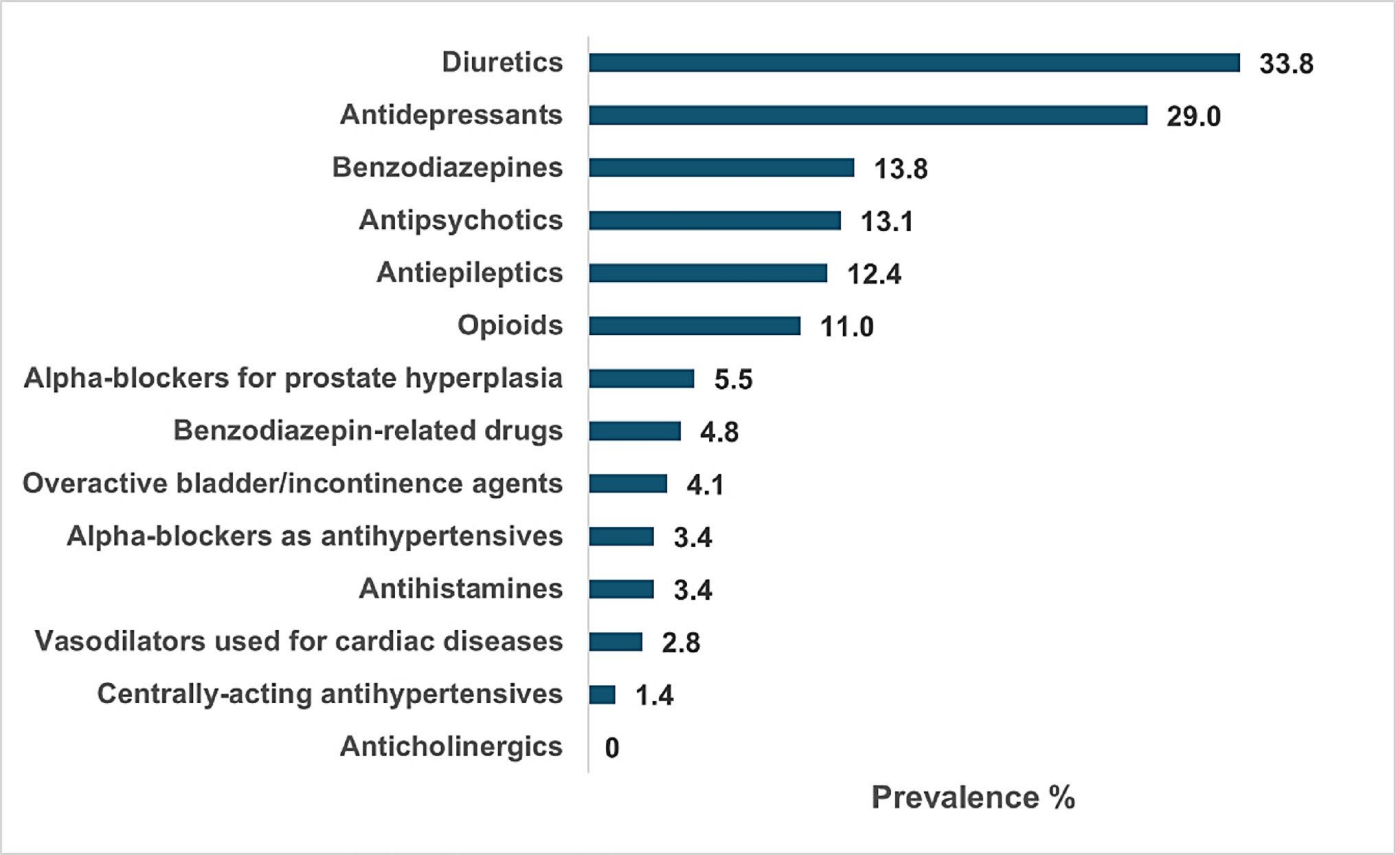
Prevalence of fall-risk-increasing drugs (FRIDs)

Of the 145 patients included in the study, 101 (70%) were taking at least one medication that increases the risk of falls (Fracture Risk Increasing Drug, FRID). The most common FRIDs were diuretics (*n* = 49), followed by antidepressants (*n* = 42) and benzodiazepines (*n* = 20). For a more detailed analysis, the individual substances within the respective substance groups were additionally recorded. In total, 239 FRID prescriptions were documented. As some patients received multiple FRIDs concurrently, the total number of FRID prescriptions exceeds the number of patients with at least one FRID. Within the diuretics class, furosemide (9.6%), hydrochlorothiazide (8.4%), and spironolactone (6.7%) were most frequently prescribed. Among antidepressants, sertraline (5.9%) and trazodone (5.0%) were the most commonly used agents (Fig. 2).

### Prevalence of fracture-associated medications

Of the 145 patients included in the study, 82 (57%) were taking at least one medication as long-term therapy that, according to Austrian guidelines, is associated with an increased risk of fracture. A total of 112 medications were identified. The most common group of substances among the medications that increase fracture risk were proton pump inhibitors (PPIs), which were documented in 58 patients (40%). Selective serotonin reuptake inhibitors (SSRIs) were also frequently prescribed, in 20 patients (14%), as were antipsychotics, in 19 (13%).

**Fig. 3:**
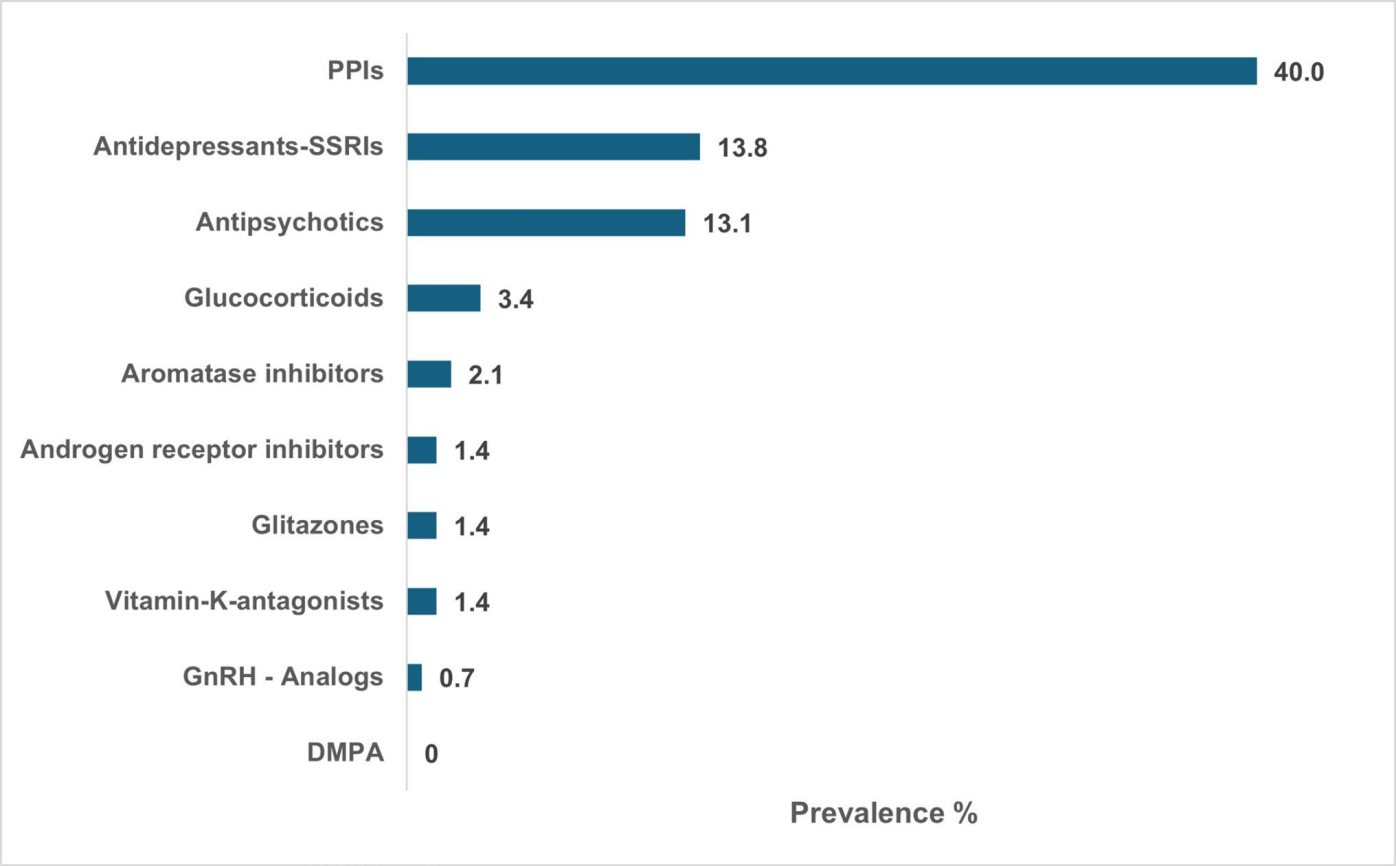
Prevalence of drug classes associated with increased fracture risk. *PPIs* proton pump inhibitors, *SSRIs* Selective serotonin reuptake inhibitor, *GnRH* Gonadotropin-releasing hormone analogs, *DMPA* Depot medroxyprogesterone acetate

Glucocorticoids (*n* = 5), aromatase inhibitors (*n* = 3), and other drug classes such as Gonadotropin-releasing hormone analogs (GnRH), androgen receptor inhibitors, or glitazones were prescribed significantly less frequently (Fig. 3).

## Discussion

This prospective observational study demonstrates a striking gap between guideline recommendations and real-world osteoporosis management in patients presenting with femoral fractures. Despite clear indications, only 7.7% of therapy-eligible patients received fully guideline-concordant osteoporosis treatment prior to the fracture.

The treatment gap observed in our cohort (78.6%) is in line with data from the SCOPE report (scorecard for osteoporosis in Europe), which estimated an average treatment gap of approximately 71% across Europe, with country-specific rates ranging from 32% to 87% [3]. Similar findings have been reported in other European cohorts, where up to 74.6% of older women at increased fracture risk remained untreated [11]. Even after major osteoporotic fractures the treatment initiation remains low. In a multinational analysis, less than 20% of previously untreated patients initiated therapy within the first year after a fracture [5, 12].

Several factors contribute to this persistent gap and can be categorized at the patient, provider, and healthcare system level. Patient-related barriers include limited disease awareness, concerns about adverse effects, and low adherence, particularly in the context of polypharmacy and advanced age [13, 14]. On the provider level, insufficient familiarity with osteoporosis treatments and underestimation of fracture risk may contribute to therapeutic inertia [15, 16]. System-level barriers include unclear responsibilities for osteoporosis management, lack of structured follow-up after fractures, and insufficient integration between inpatient and outpatient care [17].

In our cohort, the most common reason for non-concordance was the absence of pharmacological treatment despite a clear indication, highlighting a major missed opportunity for fracture prevention. Notably, even among patients with a documented diagnosis of osteoporosis, a substantial proportion remained untreated. This observation is consistent with European data showing that treatment rates remain low even after an established osteoporosis diagnosis, whereas untreated proportions exceed 90% in individuals without prior diagnosis [11]. Together, these findings suggest that the treatment gap is not solely driven by underdiagnosis but rather by insufficient implementation of guideline-based care.

Based on previous studies demonstrating a 30–50% reduction in overall fracture risk and a significant reduction in hip fractures with pharmacological osteoporosis therapy, a substantial proportion of fractures in our study population might have been preventable with timely guideline-concordant treatment [6, 18–19].

In addition to the underuse of osteoporosis therapy, a high prevalence of medications associated with increased fall and fracture risk was observed. Approximately 70% of patients were exposed to at least one fall-risk-increasing drug (FRID), and 57% received medications associated with increased fracture risk. These findings are consistent with previous studies reporting exposure rates of 50–80% in older adults with fragility fractures [20]. The frequent co-prescription of multiple FRIDs is particularly concerning, as additive effects on fracture risk have been demonstrated [21, 22].

The combination of insufficient osteoporosis treatment and high exposure to potentially harmful medications highlights a critical gap in comprehensive primary and secondary fracture prevention. Given that patients presenting with femoral fractures represent a clearly identifiable high-risk population for further osteoporotic fractures, hospitalization offers a crucial window of opportunity to initiate evidence-based osteoporosis therapy and optimize medication regimens.

The strengths of this study include its prospective design, systematic medication reconciliation, and combined evaluation of osteoporosis treatment and medication-related risk factors. However, several limitations should be considered, including the single-center design, the relatively short observation period, and potential recall bias in medication history assessment.

### Clinical implications

Our findings emphasize the need for integrated care pathways, such as fracture liaison services, and the active involvement of multidisciplinary teams, including clinical pharmacists, to detect treatment gaps and improve guideline concordance and medication safety.

## Conclusions

Patients presenting with femoral fractures exhibit a substantial gap in guideline-concordant osteoporosis treatment and a high prevalence of medications associated with increased fall and fracture risk. Systematic medication review and proactive osteoporosis management represent key opportunities to reduce fracture risks.

## Data Availability

All data supporting the findings of this study are available within the paper .
